# Laser Resection of Fibroepithelial Polyps with Digital Ureteroscopy

**DOI:** 10.1089/cren.2015.29014.sgh

**Published:** 2015-10-01

**Authors:** Scott G. Hubosky, Demetrius H. Bagley

**Affiliations:** Department of Urology, Sidney Kimmel Medical College at Thomas Jefferson University Hospital, Philadelphia, Pennsylvania.

## Abstract

Fibroepithelial polyps (FEPs) are rare benign upper urinary-tract neoplasms originating from mesodermal components of the ureteral wall covered in normal urothelial epithelium. Historically, these lesions have been treated with endoscopic, laparoscopic, or open means depending on the size, number, and morphology of the polyps. Digital ureteroscopy (DURS) offers many advantages over fiber-optic endoscopy, including superior resolution and potential ergonomic benefits, given the absence of external cameras and light cords. We describe a case involving multiple proximal ureteral FEPs treated with flexible DURS and the holmium (Ho) laser, in which the digital ureteroscope offered exceptional visualization of the FEP stalks allowing for straightforward resection and endoscopic removal.

## History and Physical Examination

A 72-year-old Caucasian man presented to the emergency room with intermittent gross hematuria and colicky right flank pain of 3 days duration without fever or nausea. Medical history was positive only for hypercholesterolemia, and surgical history was negative. The patient denied smoking history and had no known family members with cancer or nephrolithiasis. His physical examination demonstrated stable vital signs, there were no palpable masses, but he did have intermittent colicky right flank pain.

## Diagnosis

**FIG. 1.**
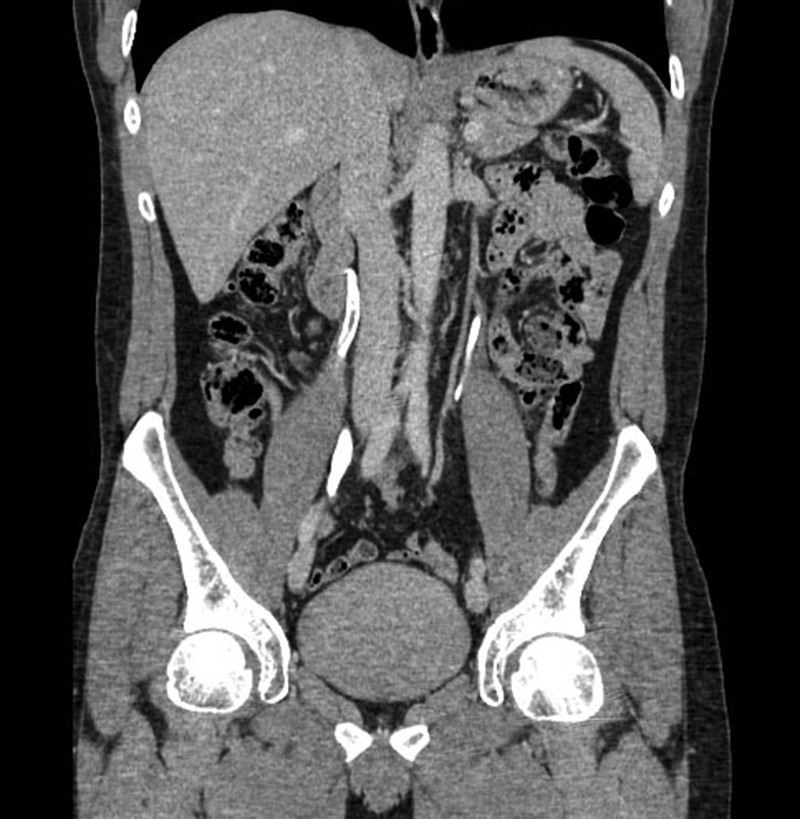
Coronal view of CT urogram shows a 3 cm linear filling defect in the right proximal ureter.

Serum creatinine was normal, and the urinalysis demonstrated gross blood without signs of infection. Urine cytology was overtly positive for numerous red blood cells, but no malignant or dysplastic cells were noted. A CT urogram (CTU) showed no urinary tract calculi but showed a 3 cm right proximal ureteral filling defect with mild hydronephrosis (Fig. 1).

## Intervention

Ureteroscopic evaluation was recommended given the CTU findings of a filling defect in the ureter along with the clinical findings of hematuria in this patient older than 70 years. The patient was counseled on the differential diagnosis, which included upper tract urothelial carcinoma (UTUC) and less likely benign etiologies, such as fibroepithelial polyps (FEPs), sloughed papilla, and blood clot secondary to upper tract hemangiomas or arteriovenous malformations. After general anesthesia was administered, rigid cystoscopy was performed with both 30° and 70° lenses. No bladder mucosal lesions were observed, and the ureteral orifices were in the orthotopic position, draining clear urine at the time. No lateralizing hematuria was noted. A right retrograde pyelogram was performed with an 8F cone-tipped catheter and ∼8 mL of 30% contrast diluted with saline at a 1:2 ratio. The image was created under fluoroscopy as the contrast was slowly injected. Findings were similar to the CTU.

**FIG. 2.**
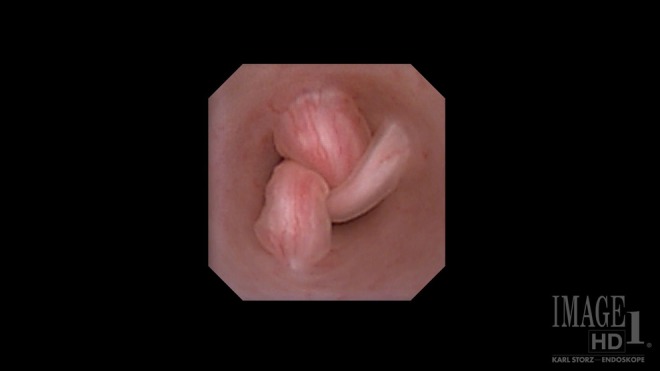
Flexible digital ureteroscopy clearly shows the stalks of three separate fibroepithelial polyps at the 12, 2, and 7 o'clock positions. The tips of the polyps are blown backwards from irrigation through the ureteroscope.

**FIG. 3.**
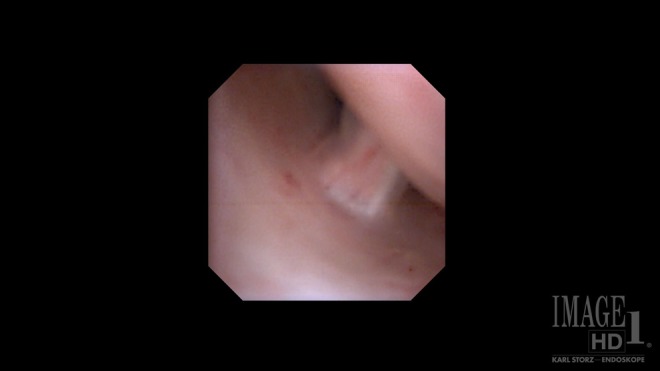
Close-up view of the polyp stalk with digital flexible ureteroscopy. Note the clear plane of separation from the normal ureteral wall (*left*) from the base of the polyp (*right*). Clear visualization allows for efficient laser resection of the stalk.

**FIG. 4.**
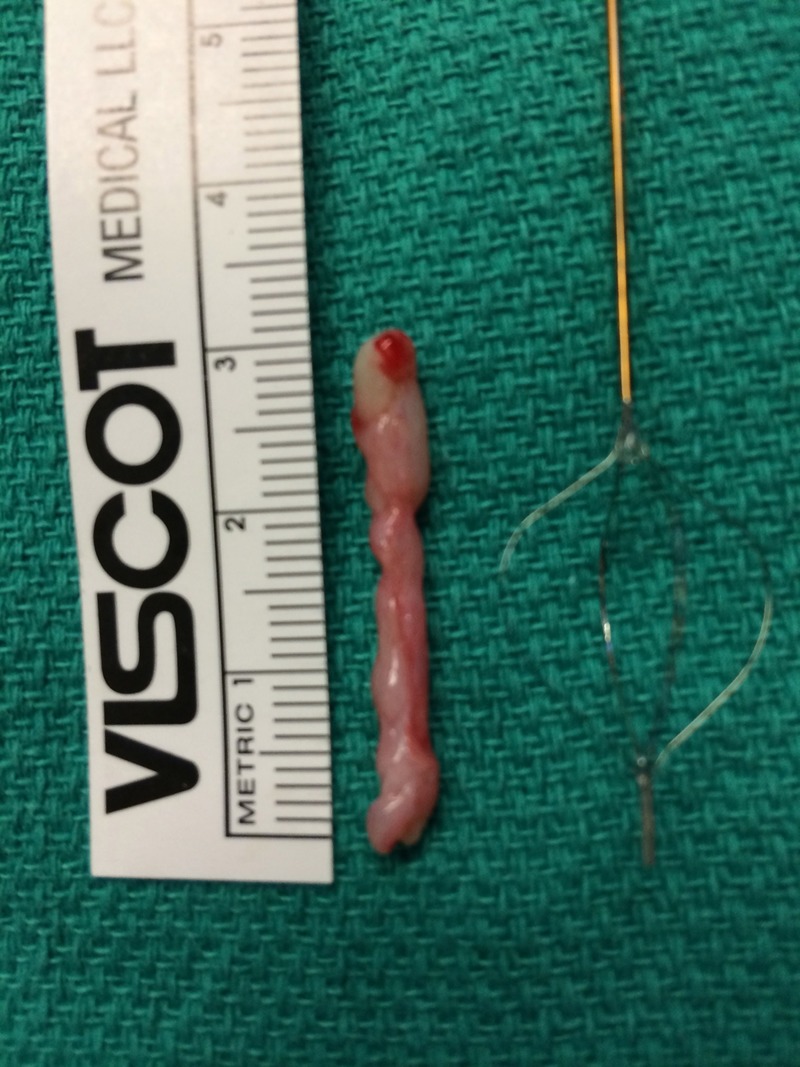
A 3 cm fibroepithelial polyp removed en bloc with a 2.4F stainless steel flat-wire basket.

Given the absence of obvious distal ureteral pathology, we inserted a flexible digital ureteroscope with constant 8.5F diameter from tip to proximal shaft, per urethra. Using no-touch technique, we carefully entered the right ureteral orifice without an initial guidewire. Inspection of the entire collecting system showed three lesions, visually consistent with FEPs, in the right proximal ureter (Fig. 2). One dominant polyp was visible and two smaller frondular polyps were also noted. Each polyp's stalk was clearly visible using the digital ureteroscope (Fig. 3). Under direct vision, a 0.038-inch stainless steel fixed core PTFE-coated wire was placed and clipped to the drape as a safety measure. After reintroduction of the flexible ureteroscope, a 365-μm laser fiber was placed and the holmium (Ho) laser was used to resect the base of each polyp at a setting of 1.2 J and 15 Hz. After the stalk of each polyp was resected away from the ureteral wall, a 2.4F stainless steel flat-wire basket was used to remove each polyp en bloc (Fig. 4). Inspection of the ureteral wall showed treatment changes from the laser, but the ureteral wall was intact. A double pigtail ureteral stent was well placed under fluoroscopy and left in place for 3 weeks. The patient went home the same day after the procedure.

## Follow-Up and Outcome

The postoperative course was uneventful except for some mild irritative lower urinary tract symptoms secondary to stent placement. The patient returned for stent removal in the office without difficulty. A follow-up visit after 4 weeks revealed a normal renal ultrasound with no residual hydronephrosis. The final pathology report demonstrated benign FEPs with no evidence of malignancy.

FEPs are extremely uncommon with just more than 200 cases reported in the literature.^1^ The largest published series reported 22 cases over a 63-year experience.^2^ In this retrospective series, FEPs were located in the proximal ureter in 59% of cases, representing the most common location in the ureter, in contrast to UTUC, which, when present in the ureter, is found most often in a distal location. Renal colic was the most common presenting symptom in the Mayo series, whereas about 20% of patients with FEPs were found incidentally. The mean polyp length was 2 cm while the range was 0.5 to 4 cm in this group. After complete endoscopic resection, with 2 years of follow-up, less than 10% of patients had recurrence.

The majority of FEPs show benign pathology. Descriptions of UTUC originating from FEPs exist in the literature,^3^ and therefore, routine pathologic examination is mandatory for complete evaluation. Given the predominantly benign nature of FEPs, it is not surprising that they often are the reported false-positive etiology of upper tract filling defects after radical nephroureterectomy for presumed UTUC, thus strengthening the argument for initial ureteroscopic evaluation in this setting.

Digital ureteroscopy has been in use for almost 10 years. With an extremely small digital camera at the distal tip of the endoscope along with light-emitting diode-powered light sources, resolution during upper tract endoscopy has arguably never been better.^4^ The initial flexible digital ureteroscopes were relatively larger in diameter than their fiber-optic counterparts. Recently, more practical smaller versions of the digital flexible ureteroscopes have been introduced with distal tip and proximal shaft diameters of 8.5F, allowing for more convenient upper tract access. Now with the best of both worlds, in terms of resolution and size, DURS enables the surgeon to clearly see upper tract pathology, including FEP stalks, thus potentially facilitating laser resection.

## Abbreviations Used

CT: computed tomography
CTU: CT urogram
DURS: digital ureteroscopy
FEPs: fibroepithelial polyps
Ho: holmium
PTFE: polytetrafluoroethylene
UTUC: upper tract urothelial carcinoma
